# Déficience Visuelle et Pathologies Oculaires À Saint-Louis, Sénégal

**DOI:** 10.48327/mtsibulletin.2021.112

**Published:** 2021-06-17

**Authors:** B. Tousignant, J. Brûlé

**Affiliations:** 1 École d'optométrie, Université de Montréal, Québec, Canada; 2 École de santé publique, Université de Montréal, Québec, Canada

**Keywords:** Déficience visuelle, Prévalence, Cataractes, Glaucome, Erreurs de réfraction, Saint Louis, Sénégal, Afrique subsaharienne, Visual impairment, Prevalence, Cataracts, Glaucoma, Refractive error, Saint Louis, Senegal, Sub-Saharan Africa

## Abstract

**Contexte:**

Il y a peu de données sur la déficience visuelle au Sénégal. Des données d'Afrique de l'Ouest estiment la prévalence de la cécité à 1,2 – 3,4% et de la déficience visuelle à 10,4 – 17,1%. IRIS Mundial (IM), un organisme non gouvernemental, contribue au développement de soins oculaires au Sénégal, avec l'Association St-Louisienne pour la vue (ASV).

**Objectifs:**

Décrire l'importance relative des causes de déficience visuelle à Saint-Louis au sein d'une population consultant pour des problèmes oculaires, afin d'aider à planifier des soins oculaires pertinents.

**Méthodes:**

Résultats d'un projet clinique de IM et de ASV (2018), compilés et analysés.

**Résultats:**

Parmi les 1944 patients (56,5% femmes) examinés, 25,7% présentent une déficience visuelle (22,5% modérée; 3,2% sévère). La myopie est présente chez 15,3% des consultants, l'hypermétropie chez 10,7%, l'astigmatisme chez 54,8% et la presbytie chez 55,8%. Les cataractes sont présentes chez 17,4% et le glaucome chez 2,5%.

**Conclusion:**

Cet état des lieux de la déficience visuelle confirme que les erreurs de réfraction, les cataractes et le glaucome sont prédominants dans une consultation d'ophtalmologie de la région de Saint-Louis. Ces résultats peuvent être considérés pour planifier des programmes de soins oculaires.

## Abstract (en)

**Background:**

Few epidemiological data describe the prevalence of blindness and visual impairment in Senegal. Data from West African studies estimate blindness prevalence between 1.2 – 3.4% and that of visual impairment between 10.4 – 17.1%. IRIS Mundial (IM) is a non-governmental organization collaborating with l'Association St-Louisienne pour la Vue (ASV) to develop eye care in Senegal.

**Objective:**

Describe the relative importance of and causes of blindness and visual impairment in a population consulting for vision problems in the Senegalese region of St. Louis, to assist in planning of relevant eye care programs.

**Methods:**

Results from eye exams carried out by a team from IM in 2018 have been complied and analyzed.

**Results:**

In all, 1944 patients were examined (56.5% female). Some 25.7% of patients presented a visual impairment (22.5% moderate; 3.2% severe). Regarding refractive error, 15.3% of patients had myopia, 10.7% had hyperopia, 54.8% had astigmatism and 55.8% were presbyopic. Clinically significant cataracts is present in 17.4% of patients and glaucoma in 2.5%.

**Conclusion:**

Our data give a glimpse of the prevalence of visual impairment and ocular disease in St. Louis, Senegal. Uncorrected refractive error, cataracts and glaucoma are present in this population and may guide the planning of relevant eye care interventions.

## Contexte

La cécité et la déficience visuelle constituent des enjeux importants de santé publique à l'échelle mondiale [3, 20, 22, 26]: leur impact sur la qualité de vie des individus de même que sur l'économie est significatif [7, 9, 17]. Parmi les quelque 285 millions d'individus affectés par une déficience visuelle (acuité visuelle de présentation < 6/18 au meilleur oeil), 90% vivent dans les pays en voie de développement. Par ailleurs, 80% des causes de cécité sont évitables, comme les erreurs de réfraction non corrigées et les cataractes. Ceci a mené à la mise sur pied de l'initiative Vision 2020: le droit à la vue de l'Organisation mondiale de la santé (OMS) et de l'*International Agency for the Prevention of Blindness* [12].

Au Sénégal, pays signataire de l'initiative *Vision 2020*, le Ministère de la santé et de l'action sociale reconnait que les pathologies responsables de la déficience visuelle sont un problème de santé publique [18]. Toutefois, peu de données sur la prévalence de la déficience visuelle et de la cécité dans le pays sont disponibles, alors que de telles données sont essentielles à la planification et au développement de services de soins oculaires.

Dans son Programme national de promotion de la santé oculaire, le Ministère de la santé et de l'action sociale du Sénégal estime à 165000 le nombre de Sénégalais atteints de cécité et à 500000 le nombre de Sénégalais atteints de déficience visuelle [18]. Des données issues d'études menées dans d'autres pays d'Afrique subsaharienne estiment la prévalence de la cécité et de la déficience visuelle à 1,2 – 3,4% et à 10,4 – 17,1%, respectivement [6, 15, 19]. Les deux premières causes de la déficience visuelle sévère et de la cécité seraient la cataracte et le glaucome; les erreurs de réfraction non corrigées représentent quant à elles la majeure partie des déficiences visuelles légères et modérées [15].

Le Sénégal s'étend sur une superficie de 196772 km^2^ dans la partie la plus occidentale de l'Afrique et compte une population de 13,5 millions d'habitants [1]. La région de Saint-Louis est située au nord du pays; ses quelques 975000 habitants sont desservis par un seul centre hospitalier, le Centre hospitalier régional de Saint-Louis, de même que quelques centres de santé où oeuvrent deux ophtalmologistes et quatre techniciens supérieurs en ophtalmologie (TSO) [18].

En novembre 2018, l'organisation non gouvernementale canadienne IRIS Mundial (IM), composée de médecins ophtalmologistes, d'optométristes et d'opticiens, s'est associée à l'Association saint-louisienne pour la vue (ASV) pour réaliser un projet clinique et épidémiologique à Saint-Louis, Sénégal.

## Objectifs

Cette étude vise à rapporter des résultats sur la déficience visuelle et l'importance relative des pathologies oculaires au sein d'une population clinique de Saint-Louis, au Sénégal. Ces données doivent contribuer à améliorer les connaissances sur les besoins en soins oculaires dans la région et pourraient appuyer la planification et le développement de soins ophtalmiques pertinents.

## Méthodes

Cette étude présente une série de cas provenant des dossiers des patients examinés par l'équipe clinique de IM et de l'ASV à Saint-Louis (échantillon de commodité). En novembre 2018, une campagne de recrutement des participants a été mise en place afin de recruter des patients ayant des difficultés à voir et ayant une situation socio-économique précaire, sans restriction d'âge. L'ASV, en collaboration avec les autorités locales, le ministère de l'éducation et des organismes communautaires locaux, a distribué quelque 2000 cartons d'invitation à consulter. La campagne de recrutement a été complétée par deux émissions radio expliquant le projet.

Les examens cliniques se sont déroulés du 22 au 27 novembre 2018 dans un centre de promotion sociale local, par une équipe clinique composée de membres d'IM et de l'ASV. L'équipe comprenait 30 bénévoles d'IM (3 ophtalmologistes, 9 optométristes, 5 opticiens, 1 médecin urgentiste, 6 infirmières et 6 autres bénévoles, logisticiens et traducteurs) et de 21 bénévoles sénégalais (1 ophtalmologiste, 1 TSO et 19 autres bénévoles, traducteurs et logisticiens). Les chirurgies nécessaires ont été effectuées au Centre hospitalier régional de Saint-Louis, par une équipe combinée de médecins et infirmières sénégalais et canadiens, sans frais pour les patients, avec le soutien financier de IRIS Mundial.

Tous les patients ont subi un examen complet incluant l'anamnèse (profil démographique, plaintes visuelles, antécédents oculaires, antécédents médicaux), l'acuité visuelle de loin (échelle de Snellen et E directionnels à 6 mètres) avec la correction disponible (acuités visuelle de présentation), les réflexes pupillaires, la réfraction objective (skiascopie et autoréfractométrie), la tonométrie (par aplanation Perkins ou par tonomètre à air sans contact) et l'évaluation du fond d'oeil faite par ophtalmoscopie directe. Les définitions de classification de déficience visuelle employées sont celles de la onzième classification internationale des maladies (CIM-011, 2018) [26]. D'autres tests étaient effectués lors de certaines conditions: la réfraction subjective (p. ex. réfraction objective non concluante ou difficile), la biomicroscopie (p. ex. acuité visuelle non améliorable par réfraction, précision d'un diagnostic d'atteinte du segment antérieur), l'évaluation du fond d'oeil sous dilatation pupillaire (p. ex. opacités des milieux oculaires, rapport excavation/papille > 0,6, tonométrie > 22 mm Hg, acuité visuelle inférieure à 6/18).

Les résultats étaient consignés sur un formulaire informatisé dérivé d'autres études épidémiologiques [4, 14, 24]. La prise de données s'est faite directement sur une tablette par les examinateurs, qui ont reçu une formation afin de les sensibiliser à l'importance d'adopter une approche uniforme et constante lors de la prise de mesures et de la tenue de dossier, de même que pour se familiariser avec l'utilisation des tablettes. Une seconde formation a été offerte in situ le premier jour au Sénégal.

Des lunettes étaient fournies sans frais aux patients en ayant besoin et une liste de patients nécessitant des soins plus avancés (p. ex. chirurgie de cataracte) a été élaborée afin que les soins soient prodigués ultérieurement, sans frais pour les patients, par un ophtalmologiste local, avec le soutien financier de IRIS Mundial.

En raison de divers facteurs, une exemption d'autorisation éthique institutionnelle a été accordée pour ce projet par le Comité d'éthique de la recherche en santé de l'Université de Montréal. Entre autres, au début de l'examen, tous les patients devaient verbalement refuser ou consentir à une éventuelle utilisation secondaire de leurs données cliniques, sous forme dénominalisée, à des fins de recherche. De plus, les auteurs n'étaient pas présents lors de la collecte de données et ont reçu les données a posteriori. Enfin, la transmission des données de IRIS Mundial aux auteurs s'est effectuée par un fichier dénominalisé, un identifiant unique ayant auparavant été attribué à chaque participant.

Des analyses statistiques des principales causes de cécité et de déficience visuelle (cataractes, glaucome, erreurs de réfraction), de pathologies oculovisuelles communes (p. ex. ptérygion) ainsi que de la distribution de lunettes ont été effectuées à l'aide du logiciel JASP v 0.10.

À des fins d'analyse, la myopie cliniquement significative a été définie comme étant une sphère (puissance réfractive sphérique) ≤ -1,0 dioptrie (D), l'hypermétropie cliniquement significative une sphère ≥ +3.0 D, l'astigmatisme cliniquement significatif une puissance réfractive cylindrique ≤ -0,5 D [13, 23] et la presbytie comme étant une addition prescrite ≥ 1,0 D.

Des cataractes étaient considérées significatives si elles atteignaient un grade 3 (sur 4 à décrire pour les non ophtalmologistes) ou plus, soit un stade chirurgical, selon l'échelle simplifiée de l'OMS [25]. Les cas de suspicion de glaucome ont été définis comme étant une pression intraoculaire ≥ 24 mmHg ou encore un rapport excavation/papille ≥ 0.7 dans l'un ou l'autre des yeux; les cas de glaucome probables ont été définis comme étant ceux présentant une pression intraoculaire ≥ 24 mm Hg couplée à un rapport excavation/papille ≥ 0,7 dans l'un ou l'autre des yeux [8, 11].

## Résultats

Un total de 1944 patients a été examiné (56,5% de femmes et 43,5% d'hommes); l'âge médian était de 52 ans (écart interquartile 34) (Tableau I). Le consentement a été obtenu pour 1944 participants (100%). Soixante-seize % (n = 1472) des patients se plaignaient de mauvaise vision au loin tandis que 68,5% (n = 1332) des patients se plaignaient de mauvaise vision au près. Quelque 65,1% (n = 1098) des patients avaient une déficience visuelle légère ou absente (acuité visuelle de présentation [AV] ≥ 6/18 au meilleur oeil), 22,5% (n = 437) avaient une déficience visuelle modérée (< 6/18 ≥ 6/60 au meilleur oeil) alors que 3,2% avaient une déficience visuelle sévère (< 6/60 ≥ 3/60 au meilleur oeil) et que 8,6% étaient aveugles (AV < 3/60 au meilleur oeil) (Tableau I). Les femmes avaient des proportions plus élevées de déficience visuelle que les hommes pour tous les niveaux.

**Tableau I:** Caractéristiques démographiques de la population à l'étude et niveaux de déficience visuelle à Saint-Louis, Sénégal (n = 1944) Demographic characteristics of study population and levels of visual impairment in St. Louis, Senegal (n = 1944)

		données disponibles (n, %)
**Sexe féminin (n, %)**	1098 (56,5)	1944 (100)
**Âge (médian, écart interquartile)**	52 (34)	1944 (100)
**Déficience visuelle**		1932 (99,3)
légère ou absente*	1266 (65,1)	
modérée†	437 (22,5)	
sévère‡	62 (3,2)	
cécité§	167 (8,6)	

* Acuité visuelle de présentation au meilleur oeil ≥ 6/18

† Acuité visuelle de présentation au meilleur oeil < 6/18 ≥ 6/60

‡ Acuité visuelle de présentation au meilleur oeil < 6/60 ≥ 3/60

§ Acuité visuelle de présentation au meilleur oeil < 3/60

**Figure. 1:**
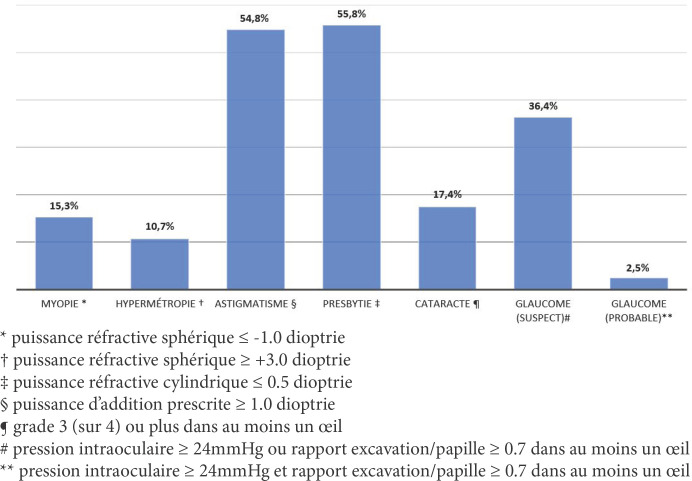
Prévalence d'erreurs de réfraction et autres causes de déficience visuelle à Saint-Louis, Sénégal (n = 1944) Prevalence of refractive error and other causes of visual impairment in St. Louis, Senegal (n = 1944)

Quelque 15,3% (n = 298) des patients présentaient, dans au moins un oeil, une myopie cliniquement significative, tandis que 10,7% (n = 208) avaient une hypermétropie cliniquement significative et 54,8% (n = 1066) avaient un astigmatisme cliniquement significatif. Cinquante-six % (n = 1084) des patients étaient presbytes (Fig. 1).

Des cataractes cliniquement significatives étaient présentes dans au moins un oeil chez 17,4% (n = 339) des patients et des cataractes bilatérales de grade 3 ou plus ont été diagnostiquées chez 6,8% des patients (n = 132).

À l'anamnèse, 5% (n = 100) des patients rapportaient avoir un glaucome. À l'examen, 36,4% (n = 708) des patients ont été classés comme suspects de glaucome et 2,5% (n = 48) ont été classés comme cas de glaucome probable. Cinq pour cent (n = 99) des patients avaient un ptérygion cliniquement significatif (> 1 mm sur la cornée) dans au moins un oeil et 4,2% (n = 82) avaient un ptérygion cliniquement significatif dans les deux yeux.

## Discussion

Cette étude rétrospective est, à notre connaissance, la première portant sur les déficiences visuelles et de leurs causes au Sénégal. Toutefois, elle comporte de nombreuses limites, principalement liées à sa méthodologie de série de cas, avec un échantillon de commodité non aléatoire, issu d'une population clinique. Les proportions rapportées doivent être interprétées avec prudence et ne peuvent être considérées comme une mesure de prévalence au niveau populationnel. En effet, les résultats rapportés peuvent être amplifiés par un biais de recrutement, puisque la campagne de recrutement a vraisemblablement mené à un échantillon de commodité qui sous-représentait les patients sains et asymptomatiques, par rapport à la population générale. Par ailleurs, il est aussi possible que les sous-catégories de cécité ou de déficience visuelle sévère soient sous-représentées dans nos résultats, car ces patients peuvent parfois éprouver plus de difficulté à accéder à des soins de santé (transport, isolement, stigmatisation, etc.). De plus, la méthodologie utilisée ne tient pas compte de certaines pathologies oculaires, endémiques en Afrique subsaharienne, qui pourraient expliquer une proportion des déficiences visuelles/cécité tels le trachome, l'onchocercose et les complications oculaires du diabète. La multiplicité de l'équipe clinique peut également entraîner un risque de variations inter-observateurs de certaines mesures (acuité visuelle, rapport excavation/papille, etc.), malgré la formation initiale donnée aux participants sur la tenue de dossier. Des études épidémiologiques comportant des méthodologies plus robuste (c.-à-d. *Rapid Assessment of Avoidable Blindness ou Rapid Assessment of Refractive Error*), comportant des échantillonnages aléatoires, pourraient pallier ces lacunes et approfondir les connaissances du portrait de la santé ophtalmique du pays.

Néanmoins, notre étude retrouve chez 25,7% des participants une déficience visuelle modérée ou sévère (22,5% et 3,2%, respectivement) et 8,6% de cécité. Ces proportions sont élevées et témoignent d'un fardeau important au sein d'une population clinique défavorisée de la région de Saint Louis. Ceci s'ajoute aux difficultés déjà existantes dans la région, étant donné que l'Afrique de l'Ouest francophone est reconnue pour avoir un accès plus difficile aux soins oculaires [19] du fait d'une densité d'ophtalmologistes inférieure à celle des pays anglophones [21].

Le fait que les femmes souffrent plus de déficiences visuelles et de cécité que les hommes, et ce pour toutes les régions du monde, est bien établi [3, 19]. Cette différence entre les sexes semble être présente chez les consultants de l'étude. Toutefois, notre échantillon étant issu d'une population clinique de commodité, ces résultats sont à interpréter de façon prudente, puisque plusieurs facteurs peuvent avoir influencé la fréquentation des femmes lors des journées cliniques (statut social, responsabilité des enfants, transport, etc.).

Les erreurs de réfraction non corrigées sont la principale cause de déficience visuelle ou de cécité dans notre échantillon. Peu de données existent sur les erreurs de réfraction non corrigées dans la région, mais Naidoo et al. les décrivent comme étant la principale cause (45%) des cas de déficience visuelle modérée à sévère au niveau populationnel en Afrique de l'Ouest [19]. Notre échantillon comporte une proportion combinée de myopie ou hypermétropie cliniquement significative de 26%, pouvant être concomitante avec la presbytie (55,8%) ou l'astigmatisme (54,8%). Les erreurs de réfraction semblent donc, au sein de notre échantillon, un enjeu important, entraînant un effet potentiellement important sur la qualité de vie, la productivité et le bien-être des individus. Compte tenu de la simplicité relative du traitement des erreurs de réfraction (orthèse visuelle) et de la rentabilité avantageuse démontrées de telles interventions [2, 10], il apparaît important d'inclure le dépistage et la prise en charge des erreurs de réfraction non corrigées dans une planification éventuelle de soins ophtalmiques dans cette région.

La deuxième cause en importance de déficience visuelle au sein de notre échantillon s'avère être la cataracte, avec une proportion de 7% de cataractes bilatérales significatives (grade 3 ou plus). Considérant que les estimations de prévalence au niveau populationnel pour l'Afrique de l'Ouest se situent à 16% [19] et que l'importance des cataractes entraîne des déficits visuels, ceci met encore davantage en lumière l'importance de développer l'offre chirurgicale dans cette région, une intervention reconnue comme une des plus rentables pour la société [5].

La proportion de participants identifiés comme ayant un glaucome probable dans notre échantillon était de 2,5% et de 36,4% pour des suspects de glaucome. Peu de chiffres fiables existent quant à la prévalence du glaucome en Afrique: la plupart des données existantes en matière de prévalence viennent de la méthodologie RAAB (*Rapid Assessment of Avoidable Blindness* [14]), une méthode validée, mais à l'utilité limitée pour le diagnostic du glaucome puisqu'une évaluation détaillée ne sera faite qu'aux patients démontrant une déficience en acuité visuelle, ce que les patients glaucomateux n'ont généralement pas avant les stades très avancés de la maladie. Bien que la population investiguée dans la Barbados Eye Study (7% de glaucome) soit de descendance ouest-africaine, [16] une comparaison semble hasardeuse en raison de l'écart des contextes et des méthodologies. Les standards actuels de diagnostic du glaucome requièrent des tests approfondis d'imagerie du nerf optique et des champs visuels, de même que leur interprétation par des experts. Il n'existe pas de traitement curatif, et les pertes de champs visuels ou d'acuité ne sont pas récupérables. Étant donné la complexité du diagnostic, du suivi et du contrôle de la maladie, il devient difficile de prioriser sa prise en charge dans un contexte à ressources limitées. Néanmoins, malgré ces difficultés, le glaucome semble représenter une priorité incontournable à moyen terme lors de la planification de services ophtalmiques dans la région, étant donnée l'importance du nombre d'individus suspects de glaucome qui nécessiteraient une investigation clinique plus poussée et des suivis ophtalmologiques réguliers.

Nos données donnent un aperçu des proportions de la déficience visuelle et de la santé oculaire au sein d'une population clinique dans la région de Saint-Louis, au Sénégal. Les erreurs de réfraction non corrigées, les cataractes et les glaucomes en sont les causes les plus fréquentes. Des études épidémiologiques additionnelles seraient nécessaires pour mieux définir le tableau épidémiologique au niveau populationnel, mais nos résultats contribuent à préciser l'importance relative de certaines conditions oculaires dans une population clinique. Ceci peut contribuer à mieux planifier le développement de programmes de santé oculaires afin de s'attaquer à ce fardeau. Dans un contexte tel que celui de l'Afrique subsaharienne où la prévalence des déficiences visuelles est la plus élevée du monde, et où la population est relativement jeune, il serait important de mettre en place des programmes de formation des ressources humaines ophtalmologiques en prévision de l'augmentation de la démographie vieillissante des années à venir.

Notre étude est un exemple encourageant de synergie entre une instance nationale locale (Association St-Louisienne pour la Vue) et une ONG étrangère (IRIS Mundial), travaillant ensemble afin d'améliorer l'accès aux soins ophtalmiques d'une population mal desservie.

## Remerciements

Les auteurs tiennent à remercier l'Association saint-louisienne pour la vue et son personnel, ainsi que l'équipe de IRIS Mundial pour leur contribution à la réalisation de ce projet.

## Conflits D'intérêts

Les auteurs ne déclarent aucun conflit d'intérêt.
